# A multi-omic machine learning approach deconstructs the role of amino acid metabolism in the immune microenvironment and prognosis of colon adenocarcinoma

**DOI:** 10.3389/fimmu.2025.1719555

**Published:** 2025-12-08

**Authors:** Hua Zhong, Jiangdong Jin, Yi Zhang, Nuo Chen, Anya Liu, Chenfei Jiang, Wenbing Zhang, Zirui He

**Affiliations:** 1 Department of General Surgery, Ruijin Hospital, Shanghai Jiao Tong University School of Medicine, Shanghai, China; 2 Department of General Surgery, Anqing First People’s Hospital of Anhui Medical University, Anqing, China; 3 Department of General Surgery, The First Affiliated Hospital with Nanjing Medical University, Nanjing, China; 4 The First Regiment, School of Basic Medicine, Fourth Military Medical University, Xian, China; 5 Department of General Surgery, Haining People’s Hospital, Haining, China

**Keywords:** colon adenocarcinoma, amino acid metabolism, multi-omic, immune infiltration, TRIP6

## Abstract

**Background:**

The clinical heterogeneity of colon adenocarcinoma (COAD) complicates patient prognosis and treatment. While metabolic reprogramming is a key driver of tumor progression, the role of histidine metabolism in the COAD immune microenvironment remains unclear.

**Methods:**

Through an integrated analysis of public single-cell and bulk transcriptomic data, we mapped the COAD cellular atlas and assessed histidine metabolism activity. A prognostic Histidine Metabolism-related Model (HRM) was constructed using an ensemble of 101 machine learning algorithms, and its biological underpinnings were explored. The function of the key gene, TRIP6, was validated via *in vitro* experiments.

**Results:**

Single-cell analysis identified epithelial cells as the hub of histidine metabolism, which remodels TME intercellular communication. The machine learning-derived HRM robustly stratified patient prognosis across a primary and two validation cohorts. High HRM scores correlated with an “infiltrated-exhausted” immune phenotype, characterized by high immune infiltration alongside elevated checkpoint expression. The key signature gene TRIP6 was identified as a driver of poor prognosis and an immunosuppressive state, and its silencing suppressed malignant phenotypes *in vitro*.

**Conclusion:**

Histidine metabolism is a critical regulator of the COAD immune microenvironment. Our prognostic model, the HRM, provides a clinically relevant tool for risk stratification, while its key mediator, TRIP6, represents a novel therapeutic target linking tumor metabolism to immune evasion.

## 1 Introduction

Colon adenocarcinoma (COAD) presents a persistent global health challenge, marked by significant incidence and mortality rates (1). The clinical trajectory of COAD is notably heterogeneous. Despite an expanding therapeutic armamentarium, the prognosis for patients with advanced disease remains unpredictable, necessitating standardized yet personalized therapeutic strategies guided by robust molecular biomarkers (2). This clinical variability points to a critical gap in our understanding of the molecular underpinnings of COAD progression, necessitating novel approaches for more precise patient stratification.

The tumor microenvironment (TME) is now understood not as a passive scaffold but as an active, complex ecosystem that critically shapes tumor development and therapeutic response. Recent studies have revealed that this ecosystem is highly organized, with spatially structured multicellular immune hubs directly influencing tumor control (3). Central to this is the tumor immune microenvironment (TIME), where the interplay between neoplastic cells and the immune system dictates patient outcomes (4). The immune contexture—defined by the type, density, and functional state of immune infiltrates—has thus emerged as a powerful prognostic and predictive biomarker, standardized as the Immunoscore, which transcends traditional staging systems (5). The profound success of immunotherapy in specific COAD subtypes, such as those with mismatch repair deficiency, further underscores the importance of the immune landscape in dictating therapeutic efficacy (6).

Emerging evidence firmly places metabolic reprogramming, a core hallmark of cancer (7), as a master regulator of this cellular interplay. Tumor-cell-intrinsic metabolic alterations create a unique metabolic landscape within the TME, initiating a form of metabolic crosstalk that profoundly influences the functional plasticity of resident immune cells, often creating distinct immunosuppressive niches. While the immunomodulatory roles of glucose and lipid metabolism are well-established, the metabolism of amino acids has gained prominence as a pivotal and complex axis in cancer immunobiology (8). However, research has predominantly focused on pathways involving tryptophan or arginine. In contrast, the contribution of histidine metabolism remains a largely uncharted area of investigation in COAD, a critical knowledge gap given the established role of metabolic plasticity in modulating therapeutic resistance (9, 10).

To deconvolve this cellular and molecular complexity, the integration of single-cell multi-omic technologies provides unparalleled analytical power (11). While bulk RNA-sequencing offers population-level insights, single-cell RNA sequencing (scRNA-seq) enables the high-fidelity dissection of the entire cellular ecosystem, as exemplified by recent comprehensive atlases of the human COAD (12). Synthesizing these multi-scale data modalities allows for the robust association of specific metabolic signatures with distinct cellular phenotypes and functional states within the TME.

Building on this conceptual framework, we posit that histidine metabolism represents a critical but overlooked node in the regulation of the COAD immune microenvironment and, by extension, patient prognosis. In this study, we perform an integrated analysis of public transcriptomic datasets to systematically characterize the landscape of histidine metabolism in COAD. From this analysis, we construct a novel prognostic signature, aiming not only to illuminate the functional role of histidine metabolism in COAD immunobiology but also to provide a new molecular tool contributing to the refined molecular classification of COAD (13) and its clinical management (14, 15).

## 2 Materials and methods

### 2.1 Data sourcing and preparation

The foundation of this study rests on publicly accessible clinical and transcriptomic data. For single-cell analyses, RNA sequencing (scRNA-seq) data from COAD tissues and their corresponding clinical details were obtained from the Gene Expression Omnibus (GEO) (16, 17). The primary dataset for constructing and validating the prognostic signature, comprising bulk RNA-sequencing profiles and patient survival information, was sourced from The Cancer Genome Atlas (TCGA) COAD project (18). To assess the model’s robustness and generalizability, two additional independent cohorts from GEO (GSE17536, GSE29621) were incorporated as external validation sets. Finally, to investigate the therapeutic relevance of a key gene, a separate cohort of melanoma patients treated with immunotherapy (GEO: GSE91061) was analyzed (19). All datasets underwent a standardized pre-processing and normalization workflow before any analysis was performed.

### 2.2 Mapping the cellular atlas of COAD

The cellular composition of COAD was charted using the Seurat package in the R environment (20). Our analytical pipeline initiated with a rigorous, multi-step quality control (QC) process to ensure only high-quality cells were retained for downstream analysis. Specifically, we filtered out low-quality cells, retaining only those with: (1) a gene count (nFeature_RNA) between 200 and 6000, (2) a total UMI count (nCount_RNA) greater than 1000, and (3) a mitochondrial gene percentage below 15%. Following this, potential doublets were addressed using the scDblFinder package. Cells exceeding a defined score threshold or classified as ‘predicted doublets’ were removed to ensure the accuracy of downstream clustering. After these QC steps, the retained expression data were log-normalized, and dimensionality was reduced linearly via principal component analysis (PCA). For visualization and clustering, the non-linear UMAP (Uniform Manifold Approximation and Projection) algorithm was applied. This process partitioned the cells into distinct populations, which were then manually annotated based on the expression of canonical marker genes to identify major cell lineages like T cells, epithelial cells, and B cells, enabling a direct comparison between tumor and adjacent tissues.

### 2.3 Gauging histidine metabolism activity

At the single-cell level, the activity of the histidine metabolism pathway was gauged using the AUCell algorithm (21, 22). This computational method yields an enrichment score (AUC score) that reflects the activity of a predefined gene set—in this case, genes related to histidine metabolism—within individual cells. By using the median AUC score from all epithelial cells as a threshold, we stratified samples into two distinct groups: a high histidine metabolism group (hmRG_high) and a low group (hmRG_low), setting the stage for comparative analyses of the TME.

### 2.4 Dissecting functional consequences of metabolic states

To understand the functional implications of these distinct metabolic states, a differential gene expression analysis was first conducted. This step identified all genes showing statistically significant changes in expression between the hmRG_high and hmRG-low groups. Subsequently, these DEGs were subjected to GO and KEGG enrichment analyses to illuminate the key biological processes and signaling pathways that characterize each metabolic phenotype.

### 2.5 Modeling intercellular communication networks

The influence of histidine metabolism on TME signaling dynamics was assessed by computationally modeling and comparing the cell-cell communication networks active in the high- and low-activity groups. The analysis quantified and contrasted the total number and strength of interactions, identified the principal sender and receiver cell types in each condition, and evaluated the information flow through critical signaling pathways (23).

### 2.6 A machine learning approach to signature construction

The creation of our prognostic signature followed a systematic, multi-stage machine learning approach. The initial step involved mining the TCGA-COAD cohort to identify a set of candidate genes robustly associated with both histidine metabolism and patient survival, using a combination of correlation, differential expression, and univariate Cox regression analyses. This refined gene set was then subjected to a rigorous training and evaluation pipeline, which systematically assessed 101 unique machine learning algorithm combinations to construct the HRM. This pipeline systematically assessed 101 unique combinations of machine learning algorithms, which were derived from a two-stage process: (1) an initial variable selection step and (2) a final model construction step. The algorithms employed included 10 distinct methods, such as Lasso, CoxBoost, Random Survival Forest (RSF), Partial Least Squares Cox Regression (plsRcox), survivalSVM, and Stepwise Cox Regression (StepCox), among others. This framework integrates multiple algorithms to evaluate and select the optimal prognostic model (24).

### 2.7 Rigorous validation of the HRM

Extensive validation was performed to confirm the prognostic robustness of the HRM. In the TCGA cohort and two separate validation sets (GSE17536, GSE29621), patients were assigned to high- or low-risk categories based on the median HRM score. Differences in overall survival (OS) between these groups were then evaluated using Kaplan-Meier curves and the log-rank test. The predictive accuracy of the signature for 1-, 3-, and 5-year survival was further quantified by calculating the area under the curve (AUC) from time-dependent receiver operating characteristic (ROC) analyses.

### 2.8 A nomogram for enhanced clinical translation

For enhanced clinical utility, a nomogram was constructed by integrating the molecular signature with key clinical variables (25). Multivariate Cox regression analysis first confirmed that the HRM was an independent predictor of survival, even when accounting for factors like patient age and N stage. All statistically independent prognostic factors were then used to build the final nomogram. The model’s reliability was evaluated with calibration curves to compare predicted versus actual survival outcomes, while its superior predictive accuracy compared to the HRM alone was demonstrated through time-dependent ROC analysis.

### 2.9 Independent prognostic power and functional insights

To verify the HRM’s utility beyond standard clinical metrics, its prognostic significance was tested within various clinical subgroups defined by age, tumor stage, and lymph node status. To shed light on the biological processes driving the observed prognostic differences, we performed functional enrichment analyses (GO, KEGG) and Gene Set Enrichment Analysis (GSEA) on the genes differentially expressed between the high- and low-risk strata.

### 2.10 A multi-angle interrogation of the tumor immune microenvironment

To systematically characterize the immunological context associated with the HRM, a comprehensive computational strategy was implemented to provide a multi-layered view of the TME (26). This approach began by inferring the proportions of diverse immune and stromal cell types within the bulk tumor using deconvolution algorithms such as CIBERTSORT and EPIC. Beyond cellular composition, the functional polarization of the TME was assessed using single-sample Gene Set Enrichment Analysis (ssGSEA), which provided a quantitative measure of the immunological activities and pathways defining each risk group. Finally, to investigate potential immune evasion tactics, this framework included a focused analysis comparing the mRNA expression levels of pivotal co-inhibitory and co-stimulatory molecules, notably PDCD1 (PD-1), CD86, and CD276 (B7-H3), between the high- and low-HRM groups (27).

### 2.11 Global TME assessment via the ESTIMATE algorithm

A global assessment of the TME’s non-malignant components was achieved using the ESTIMATE algorithm. This tool provided an Immune Score, a Stromal Score, and an overall ESTIMATE Score for each tumor sample. These scores were then used to infer the tumor’s cellular purity.

### 2.12 Predicting immunotherapy efficacy with immunophenoscore

The potential of the HRM to predict immunotherapy outcomes was evaluated using the Immunophenoscore (IPS) (28). This score, derived from a machine learning model, uses a tumor’s gene expression profile to predict its response to checkpoint blockade. We compared pre-calculated IPS values for various treatment scenarios between the high- and low-risk groups.

### 2.13 Modeling therapeutic sensitivity

We further interrogated the clinical utility of the HRM by modeling sensitivity to various therapeutic agents. This analysis drew upon the Genomics of Drug Sensitivity in Cancer (GDSC) database (29). A predictive model was constructed to estimate the half-maximal inhibitory concentration (IC50) for a wide range of chemotherapies and targeted drugs for every patient in the TCGA-COAD cohort.

### 2.14 Characterizing TRIP6’s clinical and genomic context

A comprehensive, multi-faceted investigation was focused on TRIP6, identified as a pivotal gene within our signature. The analysis encompassed its expression patterns across multiple cancer types (pan-cancer analysis), its prognostic and diagnostic performance in COAD (via Kaplan-Meier and ROC analysis), and its statistical associations with clinical stage, tumor mutational burden (TMB), microsatellite instability (MSI), and a panel of immune checkpoint genes.

### 2.15 External validation of TRIP6 in an immunotherapy setting

To substantiate the functional link between TRIP6 and treatment response, external validation was performed in an independent cohort of melanoma patients (GSE91061) treated with immune checkpoint inhibitors. Within this cohort, we compared TRIP6 expression between patients who responded to therapy and those who did not. Kaplan-Meier analysis was used to evaluate its impact on post-treatment survival.

### 2.16 Cell culture

The SW620 and RKO cell lines were purchased from the National Collection of Authenticated Cell Cultures and American Type Culture Collection and cultured in recommended media at 37°C in a humidified atmosphere containing 5% CO_2_. siRNA targeting TRIP6 and a corresponding negative control siRNA (si-NC) were purchased from RiboBio (Guangzhou, China). Detailed siRNA sequences are provided in **Supplementary Table S1**. TRIP6 mRNA expression levels were assessed by RT-qPCR to verify knockdown efficiency. All primers, designed and synthesized by Qingdao Biotechnology Company (China), are listed in **Supplementary Table S1**.

### 2.17 Cell proliferation assay

To comprehensively evaluate the impact of TRIP6 on the malignant biological behaviors of colorectal cancer cells, we conducted a series of *in vitro* functional assays. For the cell proliferation assay, 24 hours after transfection, cells were seeded into 96-well plates at a density of 2 × 10³ cells per well. At designated time points (0, 24, 48, 72, 96, and 120 hours), 10 μL of CCK-8 reagent (Vazyme, Nanjing, China) was added to each well, followed by incubation at 37°C in the dark for 2 hours. The absorbance at 450 nm was then measured using a microplate reader (A33978, Thermo Scientific) to reflect the dynamic changes in cell proliferation over time.

Cell migration ability was assessed using a wound healing assay. After transfection, cells were cultured in 6-well plates until they reached approximately 95% confluency. Uniform scratches were created in each well using a sterile 200 μL pipette tip. The wells were gently washed twice with PBS to remove detached cells and debris, and then incubated in serum-free medium. Images of the scratches were captured at 0 and 48 hours post-scratch, and the scratch width (wound gap) was quantified using ImageJ software.

Cell invasion and migration abilities were evaluated using Transwell assays, which included both migration and invasion experiments. For the upper chamber of the Transwell, 200 μL of serum-free medium containing 2 × 10^4^ treated cells were added per well. To assess invasion, the upper surface of the Transwell membrane was pre-coated with Matrigel matrix (BD Biosciences, USA), whereas for the migration assay, no Matrigel was used. The lower chamber was filled with 600 μL of medium containing 10% serum as a chemoattractant. After incubation, the cells were fixed with 4% paraformaldehyde and stained with 0.1% crystal violet (Solarbio, China). The number of cells that had migrated or invaded through the membrane was counted under a light microscope. All experiments were performed in triplicate to ensure the reliability of the results, thereby enabling a comprehensive evaluation of the effects of TRIP6 on cell migration and invasion (27, 30).

### 2.18 Statistical software and significance

All statistical analyses were conducted in R software (v4.3.3). Unless otherwise specified, a two-tailed p-value < 0.05 was considered statistically significant.

## 3 Results

### 3.1 Charting the cellular atlas of COAD

**Figure 1:**
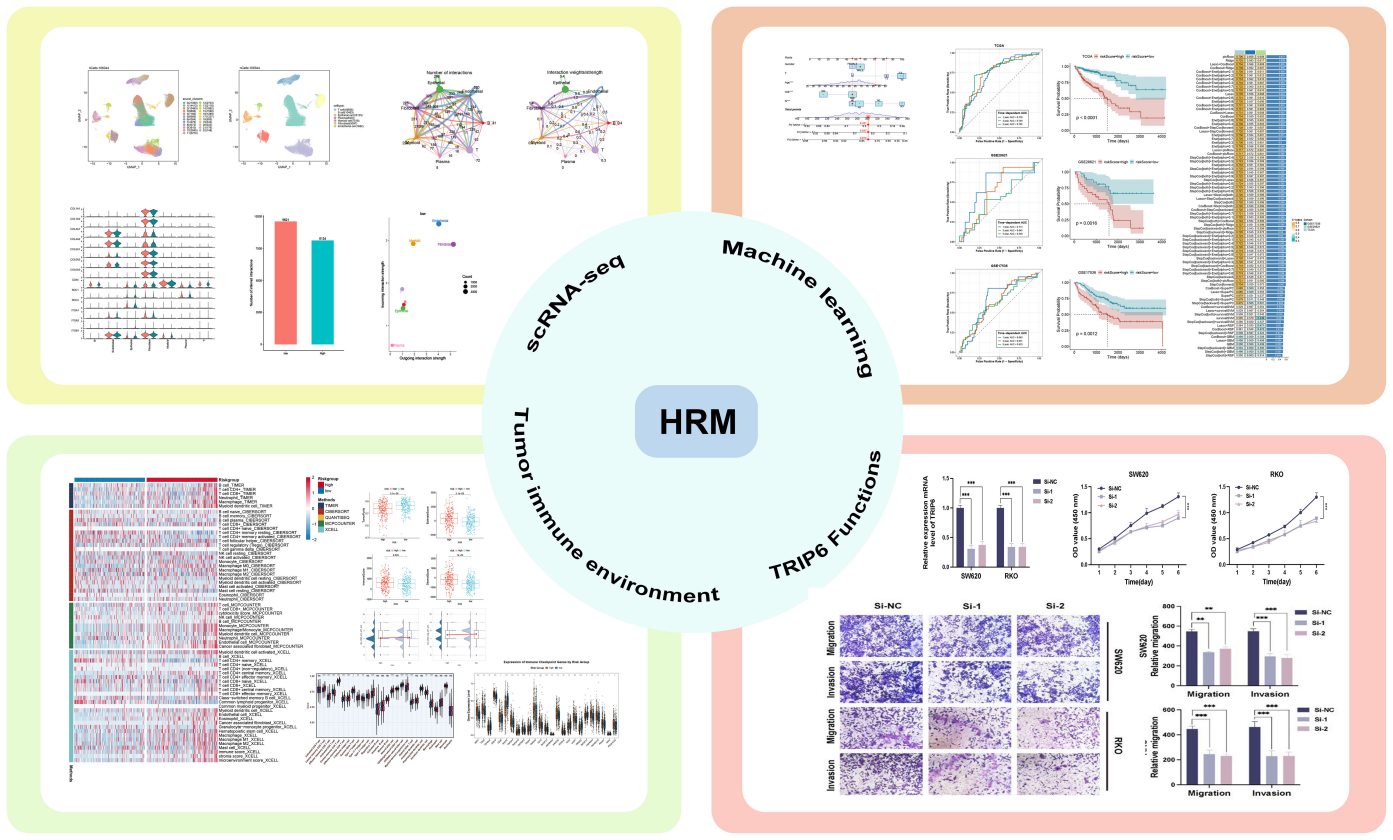
The flowchart of the research.

**Figure 2:**
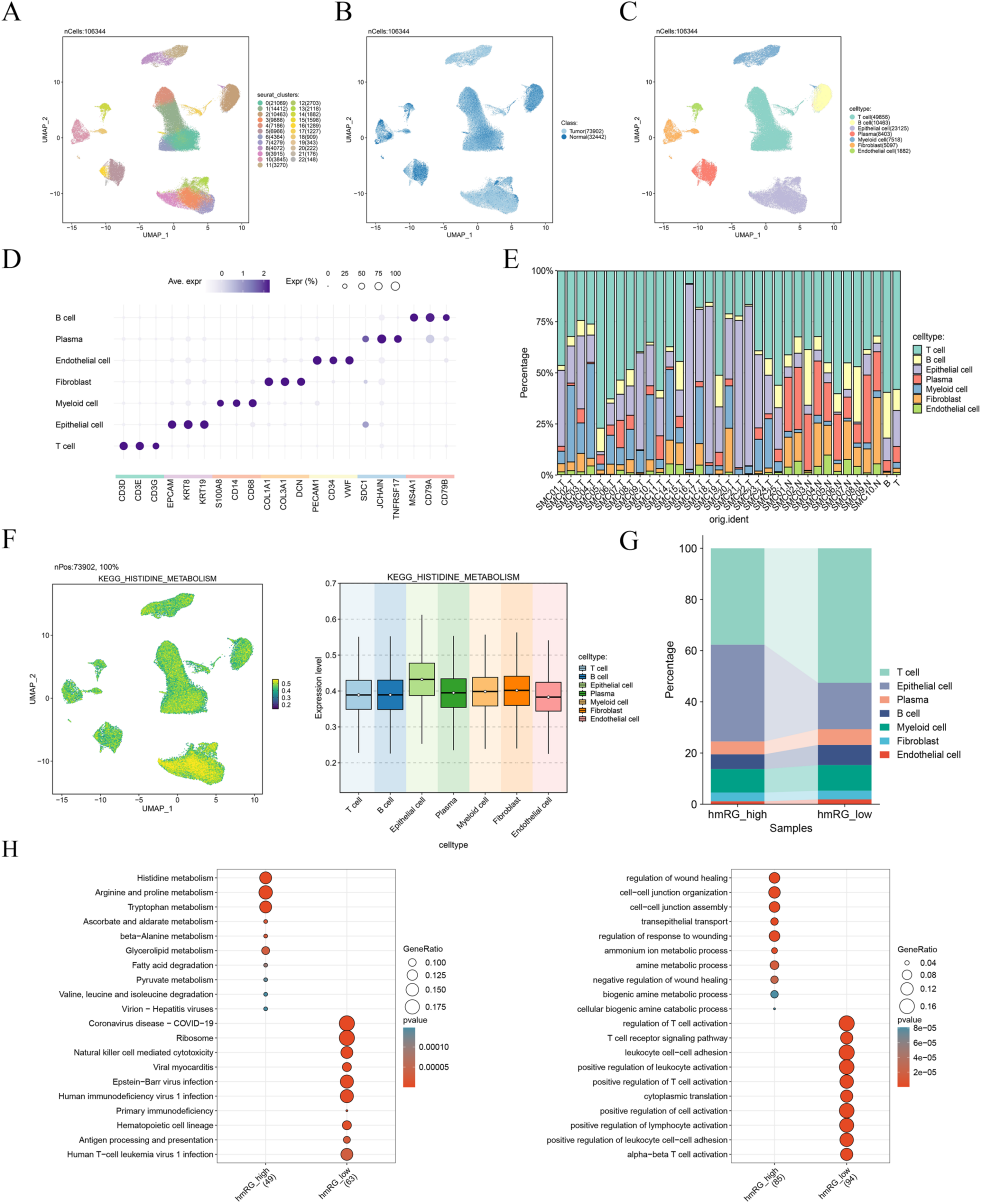
Single-cell atlas of the COAD microenvironment and histidine metabolism activity. **(A)** UMAP visualization of 23 cell clusters from COAD and adjacent tissues. **(B)** UMAP plot distinguishing cells from tumor and normal tissues. **(C)** UMAP plot of seven annotated major cell lineages. **(D)** Dot plot of canonical marker genes for cell type annotation. **(E)** Bar charts showing cellular composition of tumor versus normal tissues. **(F)** UMAP projection and violin plot of histidine metabolism activity scores (AUCell). **(G)** Cellular composition of high and low histidine metabolism groups. **(H)** Functional enrichment analysis for genes differentially expressed between high and low histidine metabolism groups.

To begin to unravel the complex cellular ecosystem of COAD, our first step was to generate a comprehensive single-cell transcriptomic map. **Figure 1** presents the flowchart of this research. We profiled both the tumor core and matched adjacent normal tissues, and after a stringent quality control pipeline, we confidently took forward a dataset comprising 106,344 individual cells for in-depth analysis. An initial unsupervised clustering immediately revealed the depth of heterogeneity within these tissues, resolving into 23 distinct clusters (**Figure 2A**). When we visualized these cells in UMAP space, a striking separation emerged between the 73,902 cells from the tumor and the 32,442 cells from the normal adjacent tissue (**Figure 2B**). This clear demarcation gave us our first glimpse into the profound transcriptomic remodeling that occurs during the development of COAD.

With this global landscape in view, our next task was to identify the specific cell types that constitute this complex microenvironment. By leveraging the expression patterns of well-established canonical marker genes, we successfully annotated these clusters into seven major cell lineages (**Figure 2C**). The tissue was a rich tapestry of immune cells, including T cells (expressing CD3D, CD3E), B cells (MS4A1, CD79A), and plasma cells (JCHAIN, TNFRSF17), interwoven with stromal components like fibroblasts (COL1A1, DCN) and endothelial cells (PECAM1, VWF), alongside myeloid cells (S100A8, CD68) and, of course, the epithelial cells (EPCAM, KRT8). The specificity of these annotations was robustly confirmed by a dot plot, which showed crisp, lineage-specific expression of our chosen markers (**Figure 2D**). As one would expect, these major cell types carved out their own distinct territories on the UMAP plot, a visual testament to their unique gene expression programs.

Beyond just identifying the players, we were keen to understand how the cellular community structure differs between a healthy colon and an established tumor (**Figure 2E**). The difference was not subtle. A deep dive into the cellular composition of each sample painted a picture of dramatic architectural reprogramming. We consistently observed that the tumor microenvironment was overwhelmingly dominated by epithelial cells and an expanded population of fibroblasts, a classic hallmark of tumorigenesis and stromal activation. In stark contrast, the adjacent normal tissues were populated by a much richer immune infiltrate, with T cells being a particularly prominent component. This compositional shift really underscores the profound dialogue between the tumor and its surroundings, reflecting the co-evolution of the malignant cells and the creation of a supportive, and likely immunosuppressive TME.

### 3.2 Epithelial cells emerge as a hub for histidine metabolism

Given the established role of metabolic reprogramming as a core hallmark of cancer, we then pivoted our analysis to explore functional states within the TME. We were particularly interested in histidine metabolism, a pathway with emerging links to cancer biology. To get a handle on which cells might be driving this metabolic activity, we utilized the AUCell algorithm to score every single cell for the expression of the KEGG “Histidine Metabolism” gene set. Projecting these activity scores onto our UMAP revealed intriguing hotspots of metabolic activity. While the pathway was not silent in other lineages, it was immediately apparent that certain populations were far more active than others (**Figure 2F**).

To put numbers to this observation and move beyond visual patterns, we directly compared the AUCell scores across our annotated cell types. The result was unequivocal. Epithelial cells displayed significantly higher and more broadly distributed histidine metabolism scores than any other cell type in the microenvironment, including the various immune and stromal populations.

To dissect this, we stratified all cells into “histidine metabolism-high” and “histidine metabolism-low” cohorts based on their AUCell scores. This partitioning immediately reinforced our earlier finding; the high-activity group was, as anticipated, overwhelmingly composed of epithelial cells (**Figure 2G**). This not only validated our cell-type-specific observation but also suggested that high histidine metabolism is a defining feature of a major subset of the epithelial compartment in COAD. With this metabolically defined population in hand, we then sought to understand its broader functional state. Enrichment analysis was performed on the differentially expressed genes between the high and low groups painted a fascinating picture of a profound metabolic overhaul (**Figure 2H**). Cells with high histidine metabolism activity were concurrently enriched for a wide array of other catabolic and anabolic pathways. Notably, we saw a strong upregulation of pathways such as ‘fatty acid degradation’, alongside other core metabolic processes. This suggests that the elevation in histidine metabolism is not an isolated event, but rather a single facet of a much larger, globally rewired metabolic engine.

### 3.3 Histidine metabolism remodels the intercellular communication architecture of the TME

Building upon our previous observations of TME heterogeneity, this study further investigated how differential histidine metabolism levels regulate intercellular communication networks. By systematically constructing and comparing cellular interaction maps between high and low histidine metabolism groups, we identified substantial remodeling of signaling architecture between the two cohorts.

**Figure 3:**
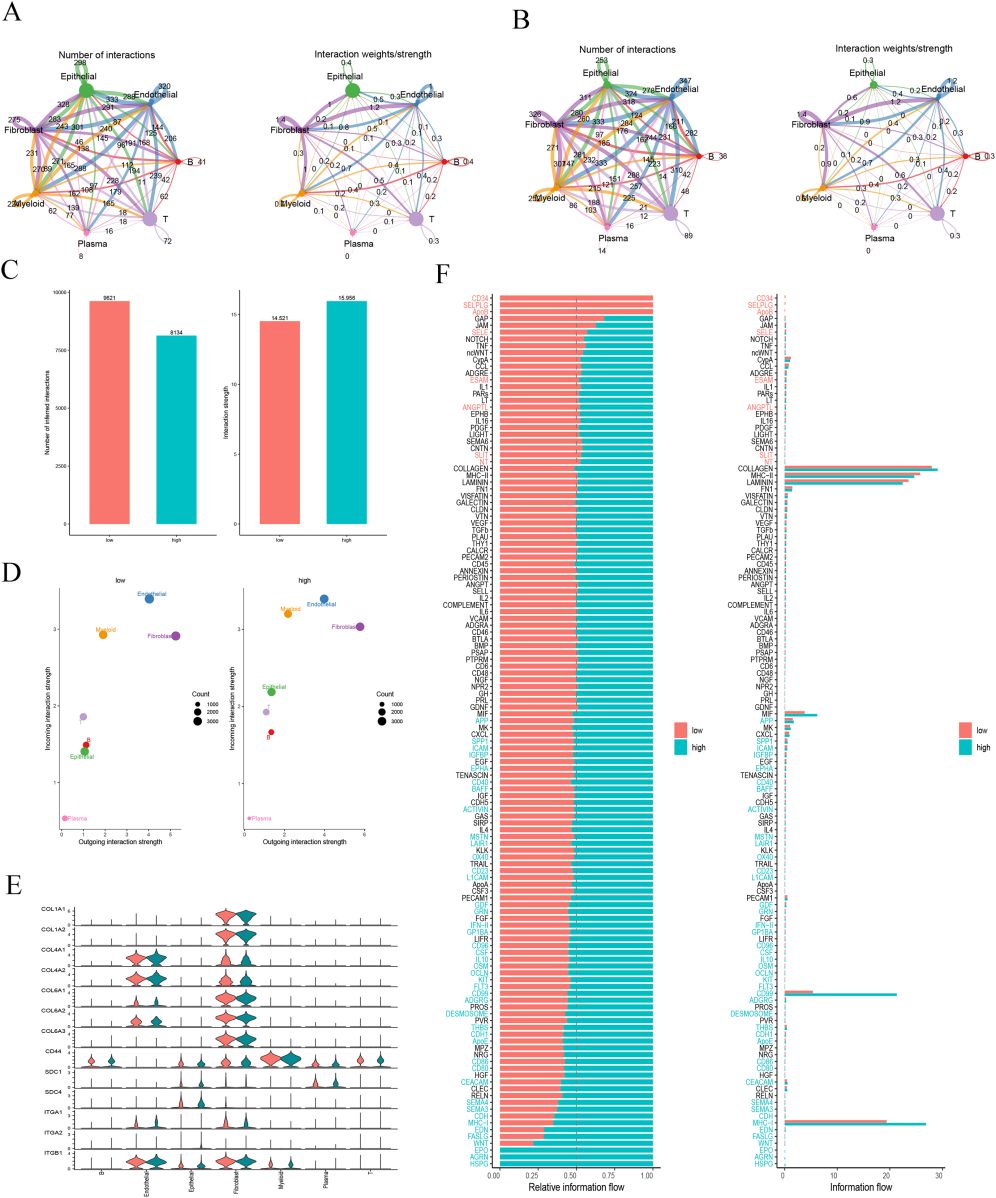
Remodeling of the intercellular communication network by histidine metabolism. **(A, B)** Cellular interaction networks in the high **(A)** and low **(B)** histidine metabolism groups. **(C)** Comparison of the number and strength of cellular interactions. **(D)** Outgoing and incoming interaction strength by cell type. **(E)** Violin plot of the COLLAGEN signaling pathway. **(F)** Comparison of information flow across major signaling pathways.

As shown in **Figures 3A, B**, the low histidine metabolism group exhibited a highly complex network of intercellular connections, particularly demonstrating dense interactions among immune cell populations. In contrast, the network structure in the high histidine metabolism group appeared notably more streamlined. Quantitative analysis further supported this observation: while the low histidine metabolism group showed a greater number of inferred ligand-receptor interactions, the high group displayed slightly stronger overall interaction strength, suggesting a shift from broad connectivity toward a more focused and potentially intensified mode of cellular communication (**Figure 3C**).

Analysis of key signal-sending and receiving cells revealed fibroblasts and endothelial cells functioning as central hubs in both groups. Notably, however, epithelial cells demonstrated significantly enhanced communication activity in the high group, showing increased involvement as both signal senders and receivers (**Figure 3D**). This suggests that elevated histidine metabolism may empower epithelial tumor cells with greater capacity to regulate the microenvironment.

Given the prominent role of fibroblasts in the signaling network, we specifically examined the highly active collagen (COLLAGEN) pathway. Our results confirmed fibroblasts as the exclusive source of multiple collagen ligands (including COL1A1 and COL1A2), while their corresponding receptors (such as CD44 and ITGB1) were widely expressed across various cell types (**Figure 3E**). This outlines a fibroblast-centered signaling axis that may promote stromal remodeling.

At the pathway level, the two groups exhibited distinct functional specialization: the low group showed more active signaling flows related to anti-tumor immunity (such as MHC-I and CD99), whereas the high group demonstrated stronger signaling intensity in pathways promoting cell adhesion and angiogenesis (**Figure 3F**). These pathway-level differences further substantiate the potential association between histidine metabolic reprogramming and functional phenotypes in the TME.

### 3.4 A histidine metabolism-based signature and integrated nomogram predict clinical outcomes in COAD

**Figure 4:**
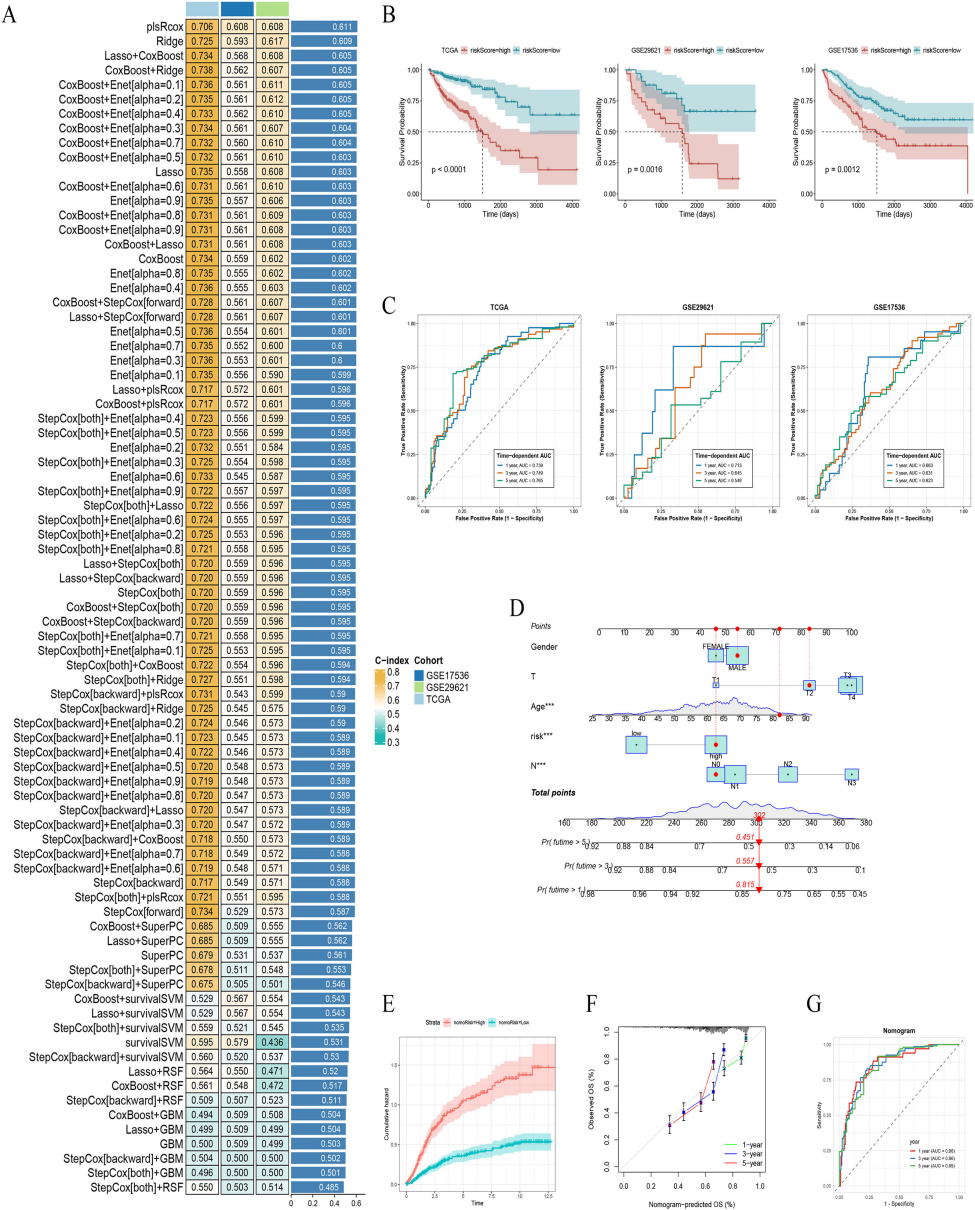
Construction and validation of the HRM prognostic model and nomogram. **(A)** C-index plot for 101 tested machine learning algorithms. **(B)** Kaplan-Meier survival curves for the HRM score in the TCGA, GSE17536, and GSE29621 cohorts. **(C)** Time-dependent ROC curves for the HRM score at 1, 3, and 5 years. **(D)** Clinical nomogram integrating the HRM score and clinicopathological variables. **(E)** Calibration curves for the nomogram at 1, 3, and 5 years. **(F)** Cumulative hazard curves for nomogram-stratified risk groups. **(G)** Time-dependent ROC curves for the integrated nomogram. The symbol *** indicates p < 0.001.

Having identified a profound metabolic signature at the single-cell level, the pivotal next step was to determine if this biological insight could be translated into a clinically meaningful prognostic tool. The challenge, of course, is to distill the complexity of a gene expression signature into a simple, robust score that can predict patient outcomes. To tackle this, we embarked on an unbiased and computationally rigorous search for the best possible predictive model. Rather than relying on a single favorite algorithm, we deployed a massive ensemble of 101 different machine learning approaches, letting the data itself guide us to the most robust method. After this exhaustive screen, one model consistently outperformed the rest: a Partial Least Squares Cox Regression (plsRcox) which demonstrated the highest C-index. From this, the HRM was born (**Figure 4A**).

The real test of any prognostic signature, however, lies in its ability to stratify real-world patient populations. We first applied the HRM score to the TCGA-COAD cohort, and the results were striking. The Kaplan-Meier curves for the newly defined HRM-high and HRM-low groups split dramatically and unequivocally (p < 0.0001), revealing a stark difference in overall survival. To rigorously test its generalizability, we challenged the HRM score with two completely independent external cohorts, GSE17536 and GSE29621. The signature held up beautifully, consistently separating patients into distinct survival trajectories in both datasets (p = 0.0012 and p = 0.0016, respectively) (**Figure 4B**). Beyond just separating groups, we needed to quantify its predictive muscle for individual patients. Time-dependent ROC analysis confirmed the model’s strong performance in forecasting 1-, 3-, and 5-year survival, solidifying its potential as a potent standalone biomarker (**Figure 4C**).

A molecular score, however powerful, often exists in the realm of research. To bridge the gap from bench to bedside, we aimed to create a tool that was not only accurate but also intuitive for clinicians. The result is a clinical nomogram, a user-friendly graphical tool that elegantly synthesizes our novel HRM score with standard clinicopathological variables—patient age, gender, and TNM stage. This allows for the calculation of a single point score that provides a personalized prediction of 1-, 3-, and 5-year survival (**Figure 4D**).

We found the nomogram to be both exceptionally accurate and highly discriminative. Calibration plots confirmed that what the model predicted was in excellent agreement with what was actually observed in patients over 1, 3, and 5 years (**Figure 4E**). Furthermore, its ability to stratify risk was profound; the cumulative hazard curves showed that patients deemed high-risk by the nomogram faced a dramatically steeper path (**Figure 4F**). The final word on its performance came from the time-dependent ROC analysis. With AUC values consistently hovering around an impressive 0.86 for 1- and 3-year survival (and 0.85 for 5-year), it was clear that this integrated model provided a far more nuanced and powerful prognostic lens than any single factor could offer alone (**Figure 4G**). By weaving our molecular discovery into the fabric of clinical practice, this nomogram represents a tangible step towards more personalized patient management in COAD.

### 3.5 The HRM score reflects opposing tumor proliferation and immune activation states

**Figure 5:**
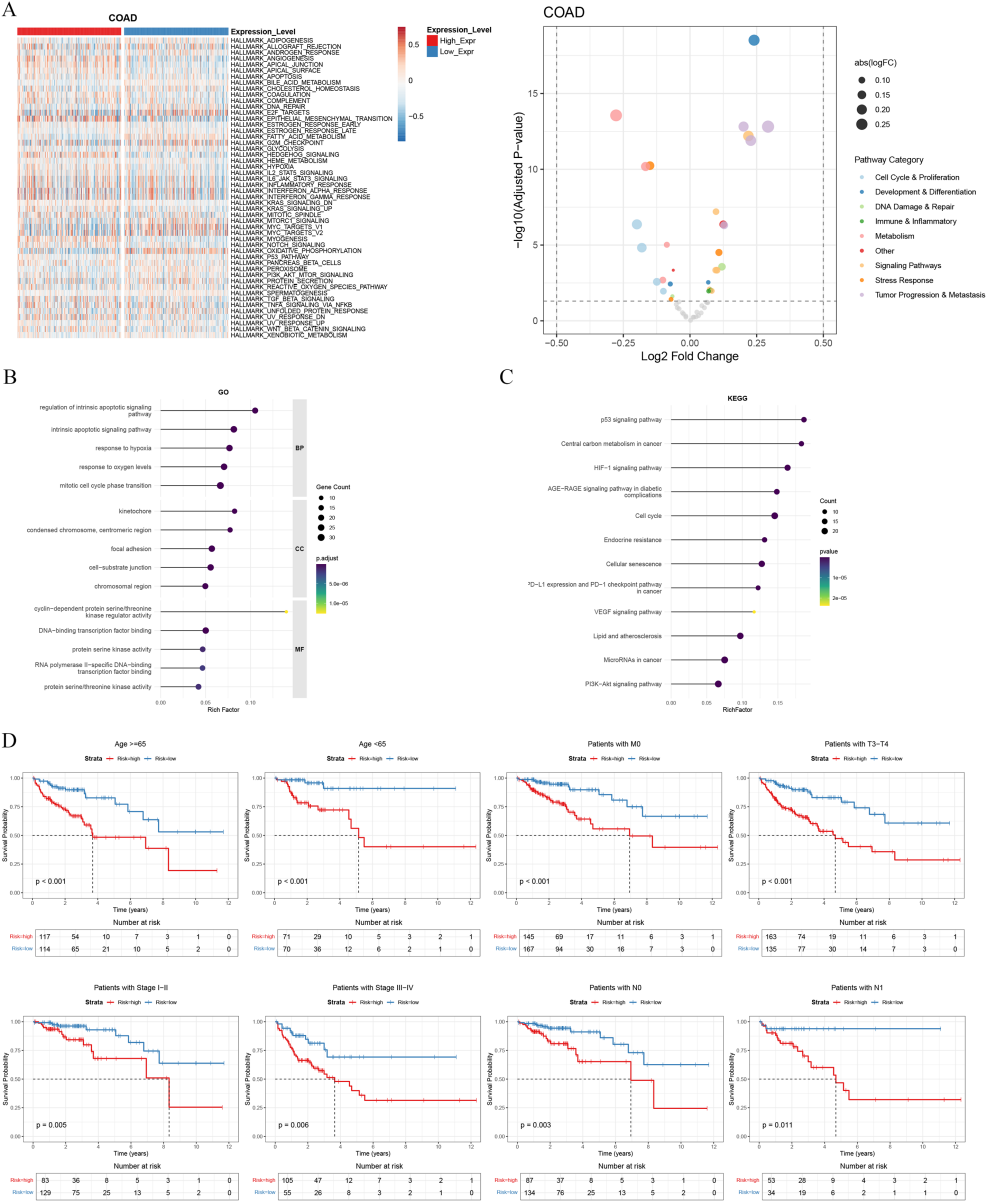
Biological pathways associated with the HRM score. **(A)** Heatmap of GSVA Hallmark pathway analysis between HRM risk groups. **(B, C)** GO **(B)** and KEGG **(C)** enrichment analysis of differentially expressed genes. **(D)** Stratified survival analysis comparing high- and low-risk groups within subgroups defined by clinical features.

To elucidate the biological mechanisms underlying the prognostic stratification by the HRM score, we performed Gene Set Variation Analysis (GSVA) using the Hallmark gene sets to compare pathway activities between the HRM-high and HRM-low risk groups (**Figure 5A**). The analysis revealed highly distinct pathway enrichment profiles. The HRM-high group, associated with poor survival, exhibited significant upregulation of pathways integral to malignant progression. Specifically, pathways related to ‘Tumor Progression & Metastasis’ were significantly enriched, suggesting that the adverse prognosis is driven by enhanced cellular proliferation activity.

Conversely, the HRM-low group was characterized by a significant enrichment of ‘Metabolism’ response pathways. Collectively, these results indicate that the HRM score encapsulates a fundamental biological dichotomy in COAD, distinguishing between tumors with a highly proliferative and aggressive phenotype and those characterized by a strong host metabolism, thereby providing a clear biological basis for its prognostic efficacy.

To further validate the biological theme identified by GSVA, we performed functional enrichment analysis on the differentially expressed genes between the risk groups. This provided a more granular, gene-level confirmation of our findings (**Figures 5B, C**). GO and KEGG analyses both revealed that genes upregulated in the HRM-high group were significantly enriched in processes fundamental to cell division, such as the ‘Cell cycle’ pathway, alongside key cancer-related cascades like ‘p53 signaling’ and ‘HIF-1 signaling’.

To further verify that the HRM score’s prognostic power was independent of standard clinical variables, we conducted a stratified survival analysis. As shown in **Figure 5D**, the HRM score retained its significant prognostic ability across the majority of clinical subgroups. Patients in the HRM-high group consistently showed poorer overall survival regardless of age, TNM stage, and tumor stage. This demonstrates that the HRM is a robust prognostic biomarker that provides additional predictive value independent of most traditional clinicopathological factors.

### 3.6 The HRM distinguishes an infiltrated but functionally exhausted immune microenvironment

**Figure 6:**
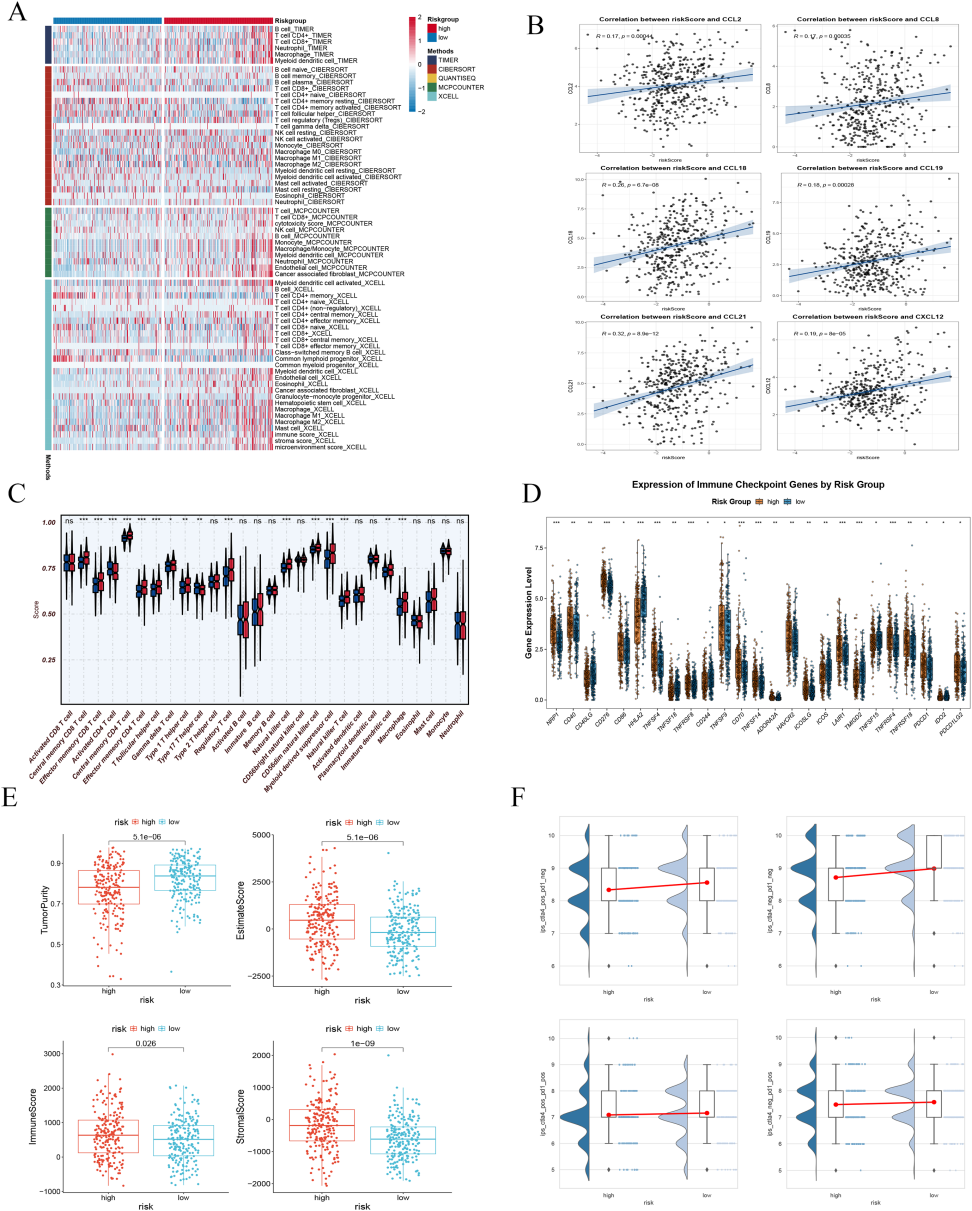
Characterization of the tumor immune microenvironment based on the HRM score. **(A)** Inferred fractions of immune and stromal cell infiltration. **(B)** Correlation between HRM score and key chemokine expression. **(C)** ssGSEA scores for immune functions between risk groups. **(D)** Expression of immune checkpoint molecules between risk groups. **(E)** Boxplot of HRM score with STROMAL score, IMMUNE score, ESTIMATE score and Tumor Purity. **(F)** Comparison of Immunophenoscore (IPS) between HRM risk groups. The meanings of the symbols are as follows: ns, not significant; *, p < 0.05; **, p < 0.01; ***, p < 0.001.

To elucidate the mechanisms underlying the prognostic significance of the HRM, we conducted an integrated analysis of the TME. Rather than being immunologically quiescent, tumors with high HRM scores displayed a notably enriched landscape of immune and stromal infiltration, as revealed by deconvolution algorithms (**Figure 6A**). This prompted us to explore the molecular drivers of such recruitment. Intriguingly, HRM levels showed a strong positive correlation with key chemokines—including CCL8, CCL19, and CCL18—suggesting a chemokine-mediated mechanism for immune cell influx (**Figure 6B**).

We next asked whether this infiltrate was functionally active or merely present. Using single-sample gene set enrichment analysis (ssGSEA), we observed that high-HRM tumors exhibited elevated immune functional scores across multiple cell types, particularly memory T cells and NK cells, implying a state of chronic immune activation (**Figure 6C**). However, such persistent immune activity often comes at a cost. In line with this, we detected widespread upregulation of immune checkpoint molecules—spanning both inhibitory and co-stimulatory members such as PDCD1, CD86, and CD276—in the high-HRM group (**Figure 6D**), pointing toward a compensatory adaptive response.

Further supporting the dense cellularity of these tumors, ESTIMATE algorithm outputs indicated that HRM scores positively correlated with Immune, Stromal, and overall ESTIMATE scores, while inversely correlating with Tumor Purity (**Figure 6E**). This composite picture—rich infiltration, high immune activity, yet broad checkpoint elevation—strongly mirrors the established molecular signature of T-cell exhaustion, a dysfunctional state that compromises antitumor immunity.

To assess the translational relevance of these findings, we employed the Immunophenoscore (IPS), a metric predictive of immunotherapy response. Consistent with a more functional and nonexhausted immune milieu, the low-HRM group displayed significantly higher IPS under both baseline conditions and in response to anti–CTLA-4 treatment (**Figure 6F**). This suggests that tumors with low HRM are better poised to benefit from immune checkpoint inhibition.

### 3.7 HRM as a predictor of sensitivity to chemotherapeutic and targeted agents

To assess the clinical potential of the HRM, we investigated its relationship with responses to various anti-cancer drugs. Our findings were striking: tumors with a high HRM score were consistently linked to resistance against a wide array of agents. This trend held true for conventional chemotherapies, such as Carmustine and Cyclophosphamide, and extended to modern targeted drugs like Afuresertib, Crizotinib, and a KRAS G12C inhibitor. Conversely, the low-HRM group emerged as the likely responders. This clear distinction suggests that the HRM could be a powerful tool for guiding treatment decisions, helping to match patients with therapies from which they are most likely to benefit (**Supplementary Figure S1**).

### 3.8 TRIP6 is a prognostic biomarker associated with malignant progression and genomic instability in COAD

**Figure 7:**
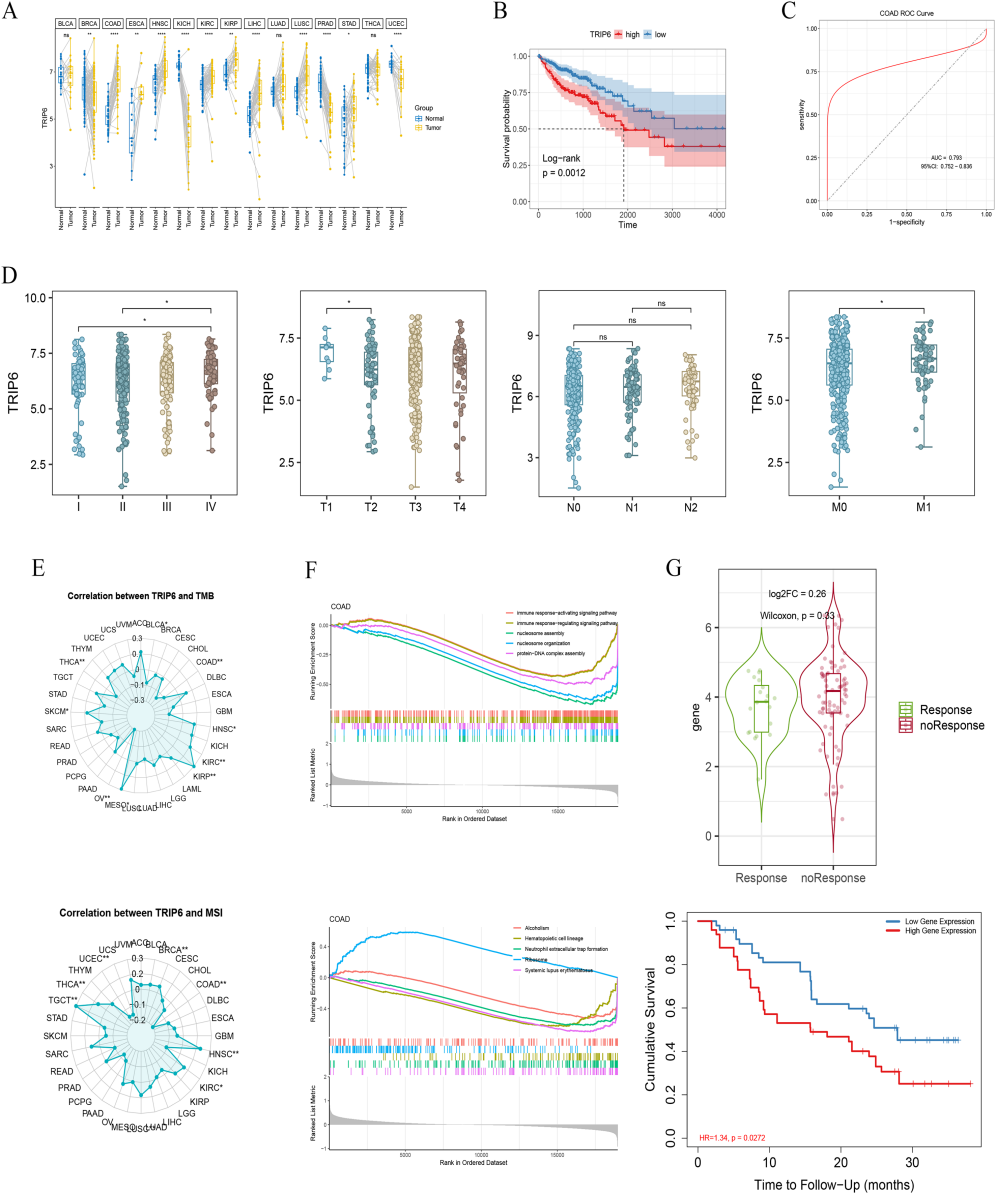
TRIP6 as a prognostic biomarker in COAD. **(A)** Pan-cancer expression of TRIP6. **(B)** Kaplan-Meier survival analysis based on TRIP6 expression. **(C)** Diagnostic ROC curve for TRIP6. **(D)** Association of TRIP6 expression with clinical T and M stages. **(E)** Correlation of TRIP6 expression with TMB and MSI status. **(F)** KEGG and GO enrichment analysis for TRIP6-high versus -low groups. **(G)** Survival analysis of TRIP6 in an external immunotherapy cohort. The meanings of the symbols are as follows: ns, not significant; *, p < 0.05; **, p < 0.01; ****, p < 0.0001.

To further validate the clinical relevance of our model, we identified Thyroid Hormone Receptor Interactor 6 (TRIP6) as a key gene within the histidine metabolism signature. Pan-cancer analysis confirmed that TRIP6 is significantly upregulated in COAD tumor tissues compared to adjacent normal tissues (**Figure 7A**). This upregulation has direct prognostic implications; Kaplan-Meier analysis revealed that patients with high TRIP6 expression have significantly poorer overall survival (p=0.0012) (**Figure 7B**). Furthermore, TRIP6 demonstrated strong diagnostic potential, with a ROC curve analysis yielding an AUC of 0.793, indicating its robust ability to distinguish tumor from non-tumor tissue (**Figure 7C**).

We next investigated the association between TRIP6 expression and clinical indicators of tumor progression. Our analysis showed a clear and significant positive correlation between elevated TRIP6 levels and advanced clinical stage. Specifically, TRIP6 expression was significantly higher in tumors of a more advanced T stage (tumor size and invasion) and in patients with distant metastasis (M1) compared to those without (M0) (**Figure 7D**). These findings strongly suggest that TRIP6 is not merely a passive marker but is actively associated with the malignant progression and metastatic potential of colorectal cancer.

Finally, to understand the broader genomic context of TRIP6, we explored its relationship with key markers of genomic instability—Tumor Mutational Burden (TMB) and Microsatellite Instability (MSI). A comprehensive pan-cancer analysis revealed that in COAD, TRIP6 expression is significantly and negatively correlated with both TMB and MSI status (**Figure 7E**). This finding indicates that tumors with high TRIP6 expression, which are associated with poorer prognosis, tend to have lower TMB and are less likely to be MSI-high. This inverse relationship suggests that the prognostic impact of TRIP6 is independent of the hypermutated phenotype often associated with favorable immunotherapy responses, linking the histidine metabolism axis to a distinct, non-hypermutated pathway of tumorigenesis.

To delve deeper into the functional consequences of TRIP6 expression, we performed enrichment analysis on TRIP6-high versus TRIP6-low patient groups. KEGG analysis revealed that high TRIP6 expression was associated with the upregulation of pathways related to protein synthesis, such as ‘Ribosome’, while GO analysis showed a significant downregulation of immune-related processes, including ‘immune response-activating signaling pathway’ and ‘immune response-regulating signaling pathway’ (**Figure 7F**). These findings suggest that high TRIP6 expression fosters a cellular state characterized by increased proliferation and a suppressed immune microenvironment. To test the clinical implication of this immune-suppressed phenotype, we examined the role of TRIP6 in an external cohort of melanoma patients undergoing immunotherapy (**Figure 7G**). In this cohort, high TRIP6 expression was significantly associated with worse overall survival (HR = 1.34, p=0.0272). Although TRIP6 expression was slightly higher in patients who did not respond to treatment compared to those who did, this trend did not reach statistical significance (p=0.33). Collectively, these results suggest that the poor prognosis associated with TRIP6 may stem from its role in promoting an immune-cold tumor microenvironment, thereby potentially limiting the efficacy of immune-based therapies.

### 3.9 Silencing TRIP6 suppresses malignant phenotypes in COAD cells

**Figure 8:**
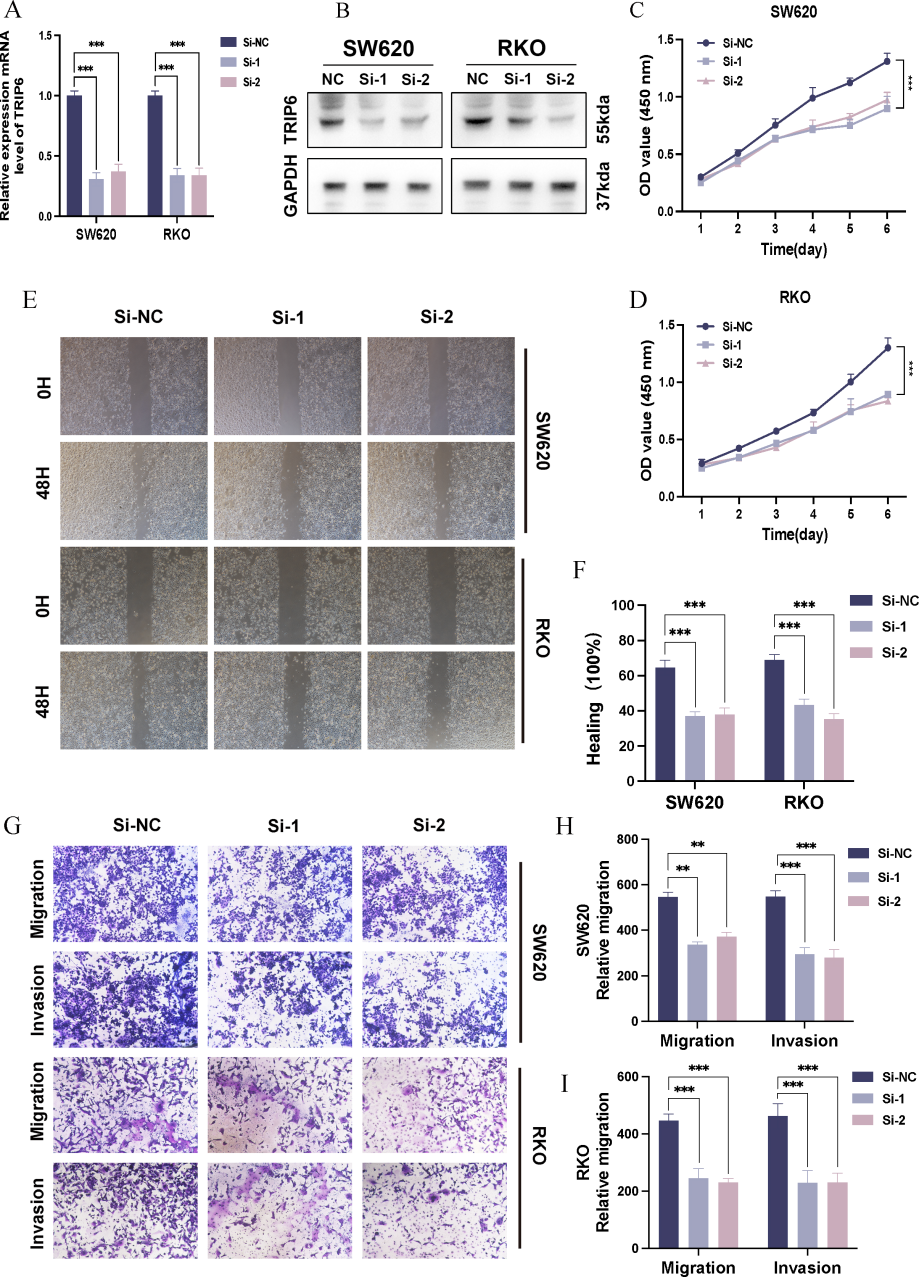
Effect of TRIP6 silencing on malignant phenotypes of COAD cells. **(A)** RT-qPCR validation of TRIP6 knockdown efficiency. **(B)** Western Blot validation of TRIP6 knockdown efficiency. **(C, D)** CCK-8 proliferation assays in SW620 **(C)** and RKO **(D)** cells. **(E, F)** Wound healing migration assay images **(E)** and quantification **(F)**. **(G–I)** Transwell invasion assay images **(G)** and quantification **(H)**, with **(I)** showing migration. The meanings of the symbols are as follows: **, p < 0.01; ***, p < 0.001.

To investigate the biological function of TRIP6 in COAD, two human COAD cell lines, SW620 and RKO, were utilized. TRIP6 expression was knocked down in these cells using specific small interfering RNA (siRNA). Quantitative real-time PCR (qRT-PCR) results demonstrated highly efficient silencing of TRIP6 at the mRNA level (**Figure 8A**). Consistently, Western blot analysis confirmed a significant reduction in TRIP6 protein expression following siRNA transfection (**Figure 8B**). Functionally, CCK-8 cell proliferation assays revealed that TRIP6 knockdown significantly inhibited the proliferation of both SW620 and RKO cells over a 120-hour period (**Figures 8C, D**). Further analysis of cell migration and invasion capabilities, assessed by wound healing and Transwell assays, respectively, demonstrated that the downregulation of TRIP6 expression markedly attenuated both migration (**Figures 8E, F**) and invasion (**Figures 8G–I**) in both COAD cell lines. In summary, these findings collectively indicate that TRIP6 plays a critical role in promoting the malignant progression of colorectal adenocarcinoma, and its knockdown effectively suppresses tumor cell proliferation, migration, and invasion *in vitro*.

## 4 Discussion

The tumor microenvironment of COAD is a highly heterogeneous and dynamically evolving ecosystem, composed of cancer cells, immune cells, cancer-associated fibroblasts (CAFs), endothelial cells, and the extracellular matrix, which plays a decisive role in tumor progression, metastasis, and treatment resistance (31, 32). Within this microenvironment, specific subtypes of CAFs secrete factors such as transforming growth factor-β (TGF-β) to establish physical barriers and induce immunosuppression, thereby hindering the infiltration of cytotoxic T cells and contributing to an “immune-excluded” or “immune-privileged” phenotype (33). Meanwhile, the accumulation of immunosuppressive cell populations—including M2-type macrophages and myeloid-derived suppressor cells (MDSCs)—further suppresses antitumor immune responses. Although immune checkpoint inhibitors (ICIs) have shown remarkable efficacy in patients with microsatellite instability-high (MSI-H) COAD, the majority of patients with microsatellite stable (MSS) COAD exhibit limited responsiveness to single-agent ICI therapy due to the immunosuppressive nature of their TME (34). Consequently, current research efforts are shifting toward combinatorial strategies, such as combining antiangiogenic agents with ICIs or targeting CAFs and reprogramming macrophages, aiming to convert “cold” tumors into “hot” tumors and enhance therapeutic responses.

Recent studies have further revealed that histidine metabolism in COAD extends far beyond its traditional role as an essential amino acid, emerging as a critical hub that links tumor cell proliferation with the regulation of the immune microenvironment (35). It not only provides one-carbon units to support nucleotide synthesis and directly drive tumor cell proliferation (36), but also contributes to the formation of an immunosuppressive microenvironment through metabolic reprogramming (37). Based on these findings, targeting key nodes in the histidine metabolic pathway is considered a promising new strategy to reverse immune suppression and enhance therapeutic responses, offering a novel approach to overcoming microenvironment-mediated treatment resistance (38).

Building on these mechanistic insights, we successfully translated our findings into a clinically applicable prognostic tool—the Histidine Metabolism-related Model (HRM), constructed via machine learning. This model demonstrates consistently robust prognostic performance across multiple independent cohorts. More importantly, immune profiling revealed that a high HRM score is associated with an “infiltrated but exhausted” immune microenvironment, characterized by progressive loss of T-cell effector function and upregulation of multiple immune checkpoint molecules (39). This state of profound T-cell exhaustion represents a key mechanism of tumor immune evasion and a major determinant of limited efficacy of single-agent immune checkpoint inhibitors (ICIs) (40). These findings not only provide a new perspective on the role of histidine metabolism in tumor immune regulation, but also suggest that combinatorial strategies targeting both metabolic pathways and immune checkpoints may hold greater therapeutic potential for tumors with impaired—but not absent—immune infiltration.

TRIP6 (thyroid hormone receptor-interacting protein 6) is a scaffold protein localized to focal adhesions and the nucleus, belonging to the PDZ domain-containing LIM protein family (41). It is primarily involved in fundamental biological processes such as cytoskeletal remodeling, integrin signaling, cell migration, adhesion, and proliferation (42). In tumor biology, TRIP6 is widely recognized as an oncogene, exhibiting aberrantly high expression in various malignancies including breast cancer, gastric cancer, and ovarian cancer (43–45). It significantly promotes tumor cell proliferation, invasion, metastasis, and chemotherapy resistance by activating pro-oncogenic signaling pathways such as Hippo-YAP and Wntβ-Catenin (46, 47).

However, the precise role of TRIP6 in tumor metabolic reprogramming, particularly in amino acid metabolism, has long remained unclear. Our study identified TRIP6 bioinformatically as a key histidine metabolism-related gene within our prognostic signature. Concurrently, our *in vitro* functional assays independently validated its role as a potent oncogenic factor, demonstrating that silencing TRIP6 significantly suppressed the proliferation, migration, and invasion of colon cancer cells.

More importantly, this study expands the purview of TRIP6 from traditional signal transduction to its potential association with the regulation of the tumor immune microenvironment. While our *in vitro* experiments focused on tumor-cell-intrinsic functions, our computational analysis of patient data revealed a strong correlation between high TRIP6 expression and an immunosuppressive phenotype, including the downregulation of immune-activating signaling pathways. This suggests that TRIP6 may be involved in shaping the TME, potentially by influencing metabolic pathways that affect immune cell function and intercellular communication, thereby promoting malignant progression. This discovery not only provides a theoretical basis for TRIP6 as a potential therapeutic target in colorectal cancer, but also unveils its potential role as a key bridge linking intrinsic tumor cell characteristics with the external immune microenvironment.

While our study provides significant insights, several limitations should be acknowledged. First, the prognostic model (HRM) was constructed and validated using retrospective data from public repositories like TCGA and GEO. Although multi-cohort validation demonstrated robustness, its clinical utility should be further confirmed in large-scale, prospective clinical trials. Furthermore, our functional validation of the key gene TRIP6 was primarily conducted *in vitro* using COAD cell lines. While these experiments confirmed its role in proliferation, migration, and invasion, these models do not fully recapitulate the complex tumor microenvironment. *In vivo* studies using patient-derived xenografts (PDX) or organoid models are needed to validate these findings within a more complex biological context.

## 5 Conclusions

This study demonstrates that histidine metabolism serves as a key regulator of the tumor immune microenvironment in colorectal cancer. We have established a clear association between this metabolic pathway and both the functional architecture of the tumor microenvironment and patient prognosis, and have developed a clinically applicable prognostic model and nomogram that provide a reliable tool for patient risk stratification and treatment decision-making. More importantly, the study reveals a unique immune phenotype associated with the model and its key driver gene, TRIP6, offering new insights into the mechanisms of immune evasion in colorectal cancer and laying a theoretical foundation for strategies targeting histidine metabolism to overcome immunotherapy resistance.

## Data Availability

The datasets presented in this study can be found in online repositories. The names of the repository/repositories and accession number(s) can be found in the article/**Supplementary Material**.
