# Supplementary material for: A multi-omic machine learning approach deconstructs the role of amino acid metabolism in the immune microenvironment and prognosis of colon adenocarcinoma
Source: Front Immunol. 2025 Dec 8;16:1719555. doi: 10.3389/fimmu.2025.1719555 (PMC12719496; doi:10.3389/fimmu.2025.1719555)
Supplement: Supplementary file 1 [file DataSheet1.docx]

Supplementary Figures


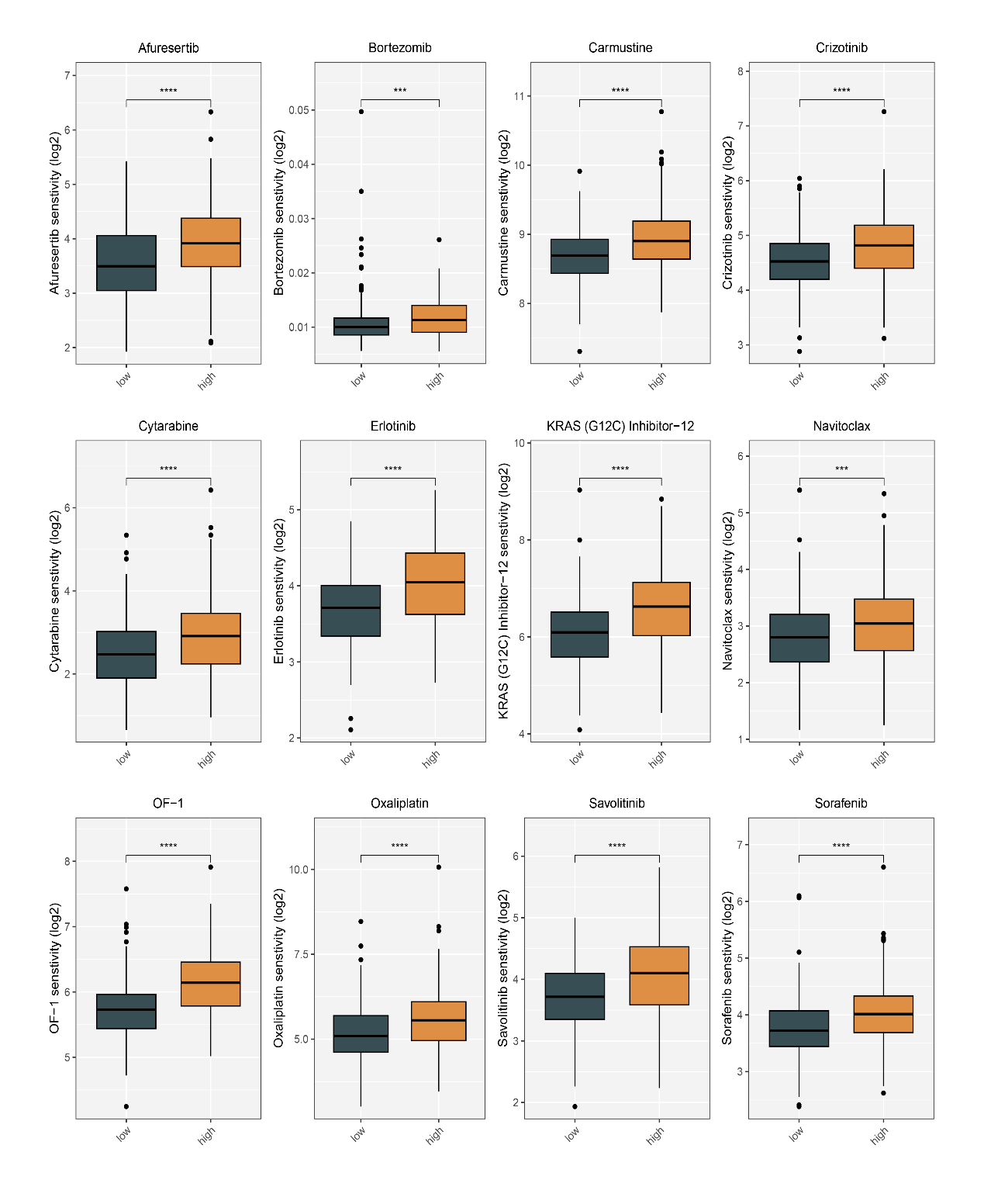


**Supplementary Figure 1. Prediction of therapeutic sensitivity by the HRM score.** (A) Predicted IC50 values for various anti-cancer drugs, compared between HRM-high and HRM-low groups.


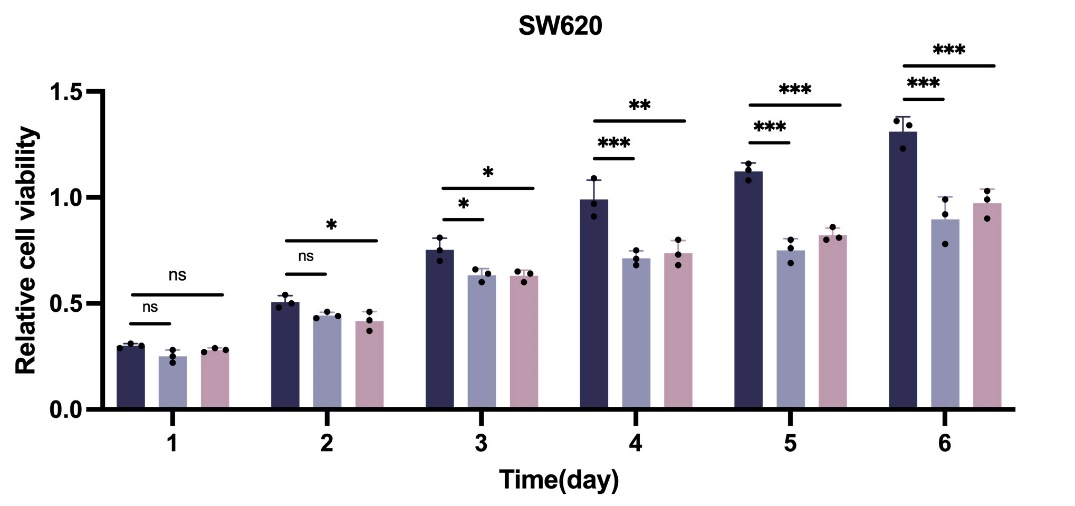


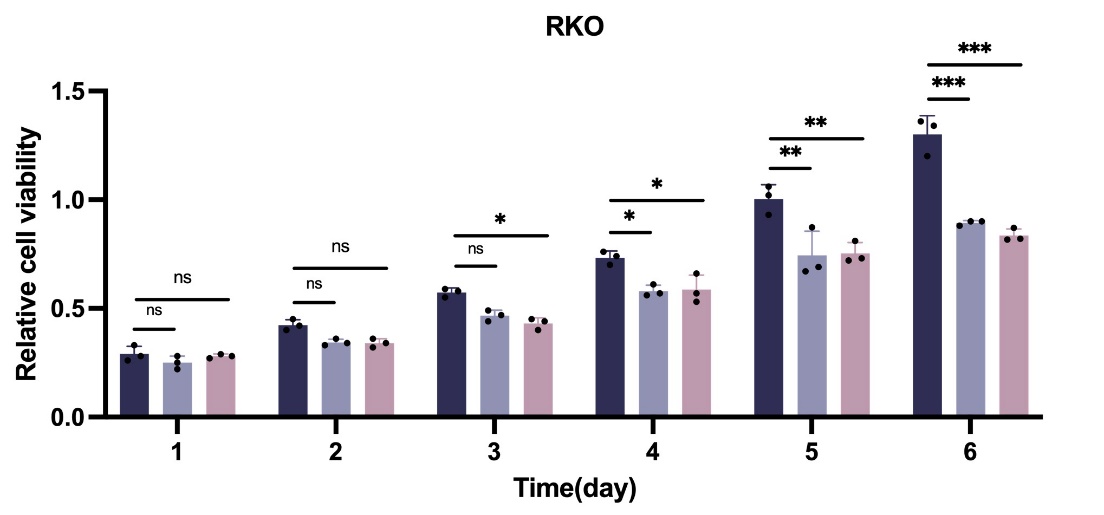


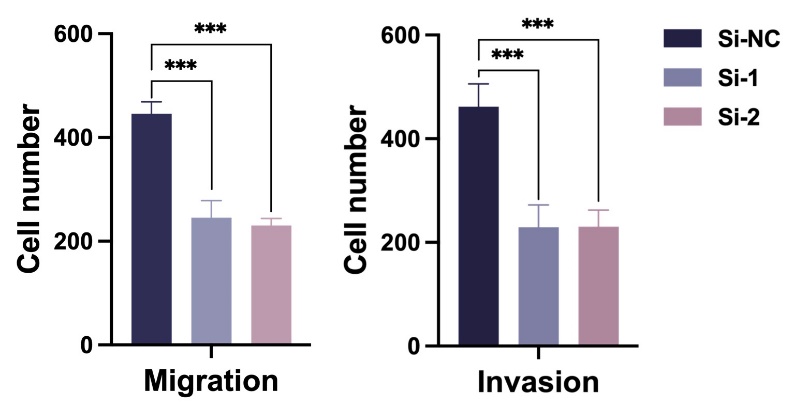


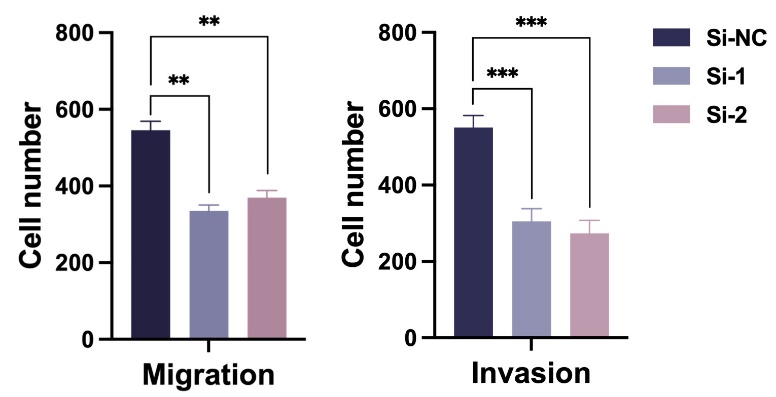


| **Oligonucleotides** | **Nucleotide sequence (5'-3')** |
| --- | --- |
| **siRNA** |  |
| Scramble control | GCUUCGCGCCGUAGUCUUA |
| Si-TRIP6-1 | GCTGCTTTGTATGTTCTACAT |
| Si-TRIP6-2 | CCTTCCATCTGAGCAGTGTTA |
|  |  |
| **Primer** |  |
| GAPDH | GGCCTCCAAGGAGTAAGACC (forward) |
|  | AGGGGAGATTCAGTGTGGTG (reverse) |
| TRIP6 | TCACGTGGGCTGCTTTGTAT (forward) |
|  | GGATCTGGCTCGTAGCATCC (reverse) |
|  |  |

**Supplementary Table 1. Oligonucleotides used in research**
